# Matrix-Assisted Laser Desorption Ionization–Time-of-Flight Mass Spectrometry with Time-of-Flight Peak Analysis for Rapid and Accurate Detection of Group B Streptococcus in Pregnant Women

**DOI:** 10.1128/spectrum.01732-21

**Published:** 2022-04-18

**Authors:** Daiki Tanno, Kyoichi Saito, Kazutaka Ohashi, Masahiro Toyokawa, Yukio Yamadera, Hiroki Shimura

**Affiliations:** a Department of Clinical Laboratory, Fukushima Medical University Hospital, Fukushima, Japan; b Department of Laboratory Medicine, School of Medicine, Fukushima Medical University grid.471467.7grid.411582.bgrid.471467.7grid.411582.bgrid.471467.7 , Fukushima, Japan; c Department of Clinical Laboratory Sciences, School of Health Sciences, Fukushima Medical University grid.471467.7grid.411582.bgrid.471467.7grid.411582.bgrid.471467.7 , Fukushima, Japan; University of Cincinnati

**Keywords:** *Streptococcus agalactiae*, GBS screening in pregnant women, MALDI-TOF MS

## Abstract

Severe infections in neonates caused by Streptococcus agalactiae, Group B Streptococcus (GBS), are often associated with GBS transmission from their mothers during labor or birth. Hence, it is necessary to develop a universal method for screening vaginal–rectal GBS colonization in pregnant women worldwide. A subculture of vaginal–rectal swabs using a selective enrichment broth and an agar plate is conventionally recommended for GBS screening. However, infants born to mothers who are GBS negative on subculture sometimes contract GBS infections. Therefore, we developed another method with high sensitivity for GBS screening. A total of 178 vaginal–rectal swabs from pregnant women were inoculated into the enrichment broth, of which 126 were suspected of containing GBS due to the change in the color of the broth. The subculture results were positive for GBS in 34 (27.0%) swabs. Each broth was then analyzed using matrix-assisted laser desorption ionization–time-of-flight mass spectrometry (MALDI-TOF MS). Analysis of the TOF peaks specific to GBS revealed 45 (35.7%) swabs as GBS positive. Of the 11 GBS positive samples on TOF peak analysis but negative on subculture, S. agalactiae gene targets were detected through PCR in 4 samples. MALDI detection with analysis of peaks of TOF (MDAPT) can detect GBS directly from cultured broth with high sensitivity. MDAPT can be an alternative method for GBS screening in pregnant women and contribute to the prevention of severe GBS infectious diseases in neonates.

**IMPORTANCE** As previously reported, 10%–30% of pregnant women carry Streptococcus agalactiae, Group B Streptococcus (GBS), in their vagina or rectum, and approximately 50% of them vertically transmit GBS to their neonates during labor or birth. Moreover, 1%–2% of the GBS-transmitted neonates develop severe GBS infectious diseases, which have a mortality rate of 19.2% in a preterm infant and 2.1% in a full-term infant. Hence, universal screening for GBS colonization in pregnant women is conducted worldwide using the subculture procedure; however, infants born to GBS negative mothers sometimes contract GBS infections. Therefore, other laboratory techniques are required for detecting GBS more accurately. The proposed method “MALDI detection with analysis of peaks of TOF (MDAPT)” detects GBS directly from cultured broth with high sensitivity. Therefore, it can be an alternative method for GBS screening in pregnant women, thereby contributing to the prevention of severe GBS infectious diseases in neonates.

## INTRODUCTION

Streptococcus agalactiae, Group B Streptococcus (GBS), are Gram-positive chained cocci that often cause severe infections, such as sepsis and meningitis, in neonates. GBS infectious diseases in neonates are clinically categorized into two syndromes: early-onset disease (EOD) and late-onset disease (LOD) (1). EOD is a GBS infection within the first week after birth caused by vertical transmission from GBS-carrying mothers to their neonates through the genital tract during labor and birth. LOD is related to GBS infection after 7 days of age, which includes both vertical and horizontal transmission, the latter of which involves other individuals and the environment. As reported in the United States, 10%–30% of pregnant women host GBS in the vagina or rectum (2, 3), and vertical transmission occurs in approximately 50% of these women. As a result, 1%–2% of the neonates of these GBS-carrying women will develop EOD (4, 5), which has a mortality rate of 19.2% in a preterm infant and 2.1% in a full-term infant (6).

To prevent EOD, the Centers for Disease Control and Prevention (CDC) mentioned in 2010 the necessity of universal screening for vaginal GBS colonization in pregnant women (1), and the CDC 2010 guidelines were revised with the American College of Obstetricians and Gynecologists (ACOG) in 2019 (7). However, the conventional method using a selective enrichment broth, which is recommended worldwide for the detection of GBS, occasionally results in false-negative results. In an epidemiological survey in the United States in 2019, 83% of the mothers of neonates who developed EOD had negative GBS screening results before childbirth (6). Therefore, other laboratory techniques for GBS detection have been required and developed to reduce false negatives and to decrease EOD incidence, including improvement of the enrichment broth (8, 9) and nucleic acid amplification tests (NAATs) such as PCR (10–12). Those studies also indicate that some of the techniques contribute to a reduced workload, costs, and turnaround times on the GBS screening in pregnant women.

Matrix-assisted laser desorption ionization–time-of-flight mass spectrometry (MALDI-TOF MS) has been used in clinical laboratories worldwide to identify pathogenic microbes isolated clinically. Moreover, MALDI-TOF MS reduces the turnaround times for diagnosing bloodstream infections by identifying pathogens directly from blood culture media with the use of certain reagents (13–15). Among such reagents, the rapid BACpro II (Nittobo, Fukushima, Japan) has been found to have a higher identification rate compared with others (16). We considered it applicable in direct GBS detection from cultured enrichment broth.

In this study, at first, we evaluated the GBS detection rate using BACpro II-assisted MALDI-TOF MS (direct MALDI detection) and compared it with the conventional subculture method. Then, we confirmed the additional analysis of TOF peaks specific to GBS turned out useful in improving the accuracy of GBS detection. Here, we propose a method, “MALDI detection with analysis of peaks of TOF (MDAPT),” which detects the existence of GBS by analyzing the TOF peaks, as a novel strategy for rapid and accurate GBS detection.

## RESULTS

### Change in color of inoculated enrichment broth.

Of the 178 vaginal–rectal swabs inoculated into Kyokuto enrichment broth, 126 had broths that changed from purple to yellow, whereas those of the 52 remained purple or changed to an intermediate color. From the 126 yellow-broth samples, 34 GBS were detected by the subculture method as described below. On the other hand, the 52 non-yellow-broth samples were also subcultured and identified using MALDI-TOF MS from each isolated colony, but no GBS were detected.

### GBS detection rates of each method.

Of the 178 vaginal–rectal swabs inoculated to enrichment broth, the subculture method and direct MALDI detection were conducted for the 126 yellow-broth samples. The GBS detection rate was 27.0% (34/126) for the subculture method and 19.0% (24/126) for the direct MALDI detection (Table 1). When considering the results of the subculture method as a reference, the sensitivity and specificity for GBS detection using the direct MALDI detection were 70.6% (24/34) and 100% (92/92), whereas the PPV and NPV were 100% (24/24) and 90.2% (92/102), respectively.

**TABLE 1:** Evaluation of direct matrix-assisted laser desorption ionization detection compared with subculture method

Direct MALDI detection	Subculture method		
	Positive	Negative	Total
Positive	24	0	24
Negative	10	92	102
Total	34	92	126

The bacterial identification results using the Microflex LT for the 10 samples that were GBS positive on the subculture but negative on direct MALDI detection were as follows: Four samples with S. agalactiae (1.757–1.960, MALDI identification score); one sample each with Enterococcus faecalis (1.737), *S. anginosus* (1.962), *S. lutetiensis* (1.850), and *S. salivarius* (1.873); and two samples with no bacteria identified with the reliable MALDI score for species (≥2.000) or genus identification (≥1.700) (Table 2).

**TABLE 2:** Results of discrepancies between the subculture method and direct matrix-assisted laser desorption ionization detection

No.	MALDI identification	Reliability score value	GBS detection by the subculture method	Bacteria other than GBS detected by the subculture method
40	Enterococcus faecalis	1.737	Positive	Lactococcus garvieae Enterococcus faecium Enterococcus casseliflavus
47	Streptococcus agalactiae	1.960	Positive	Staphylococcus hominis
72	No reliable identification	1.683	Positive	Staphylococcus epidermidis Enterococcus avium
89	Streptococcus agalactiae	1.757	Positive	Staphylococcus epidermidis Staphylococcus haemolyticus *Bacillus* spp.
121	Streptococcus anginosus	1.962	Positive	None
154	Streptococcus lutetiensis	1.850	Positive	Lactobacillus gasseri Staphylococcus lugdunensis
157	Streptococcus salivarius	1.873	Positive	Enterococcus faecalis Enterococcus avium Enterococcus mundtii
167	Streptococcus agalactiae	1.887	Positive	Enterococcus faecalis Enterococcus avium
172	No reliable identification	1.680	Positive	Streptococcus lutetiensis Staphylococcus epidermidis
176	Streptococcus agalactiae	1.758	Positive	None

### Analyses of GBS-specific TOF peaks in each sample.

Following direct MALDI detection, the TOF peaks of 126 yellow-broth samples were analyzed. Subsequently, 45 samples showed one or more of Peak 1 to 4 within 4 *m/z* and intensities of more than 6,000 AU. Of these 45 samples, 24 had already been evaluated as GBS positive on direct MALDI detection; however, the remaining 21 samples were reevaluated as GBS positive on MDAPT, which resulted in the GBS detection rate of 35.7% (45/126) for MDAPT (Table 3). The sensitivity and specificity for GBS detection with MDAPT were 100% (34/34) and 88.0% (81/92), whereas the PPV and NPV were 75.6% (34/45) and 100% (81/81), respectively.

**TABLE 3:** Results of re-analysis using matrix-assisted laser desorption ionization detection with analysis of peaks of time-of-flight (MDAPT) and nucleic acid amplification test (NAAT)

No.	Index	Peak 1	Peak 2	Peak 3	Peak 4	Subculture method	Direct MALDI detection (MALDI score)	MDAPT	NAAT	
		6734.41 m/z	6937.95 m/z	7963.55 m/z	8200.27 m/z				*cfb*	*dltS*
40	m/z		6939.318			+^*a*^	Enterococcus faecalis (1.737)	+	+	+
	Intensity (AU)		6263							
	S/N		5							
47	m/z	6731.641	6937.063		7959.717	+	Streptococcus agalactiae (1.960)	+	+	+
	Intensity (AU)	6882	6224		13864					
	S/N	8	7		23					
72	m/z	6735.600	6939.603			+	No reliable identification (1.683)	+	+	+
	Intensity (AU)	8471	8835							
	S/N	9	10							
89	m/z	6735.617	6940.159	7964.134	8201.257	+	Streptococcus agalactiae (1.757)	+	+	+
	Intensity (AU)	12722	14212	11824	7172					
	S/N	9	11	14	7					
121	m/z	6376.379	6938.244			+	Streptococcus anginosus (1.962)	+	+	+
	Intensity (AU)	6360	6751							
	S/N	4	5							
154	m/z			7963.667		+	Streptococcus lutetiensis (1.850)	+	+	+
	Intensity (AU)			10462						
	S/N			8						
157	m/z	6735.487	6939.556	7964.148	8201.312	+	Streptococcus salivarius (1.873)	+	+	+
	Intensity (AU)	8347	8095	11142	6305					
	S/N	6	6	19	8					
167	m/z	6734.613	6938.521	7962.705		+	Streptococcus agalactiae (1.887)	+	+	+
	Intensity (AU)	8773	9192	8248						
	S/N	8	10	13						
172	m/z		6941.019	7965.046		+	No reliable identification (1.680)	+	+	+
	Intensity (AU)		6886	12315						
	S/N		3	16						
176	m/z	6737.348			8203.266	+	Streptococcus agalactiae (1.758)	+	+	+
	Intensity (AU)	12860			8995					
	S/N	10			10					
20	m/z			7965.414		−	Streptococcus gallolyticus (2.092)	+	−	+
	Intensity (AU)			7001						
	S/N			9						
25	m/z			7964.603		−	No reliable identification (1.674)	+	+	+
	Intensity (AU)			7195						
	S/N			8						
28	m/z			7966.440		−	Streptococcus alactolyticus (1.879)	+	−	−
	Intensity (AU)			7303						
	S/N			12						
49	m/z	6730.703				−	Streptococcus anginosus (1.942)	+	−	−
	Intensity (AU)	8757								
	S/N	6								
56	m/z	6731.595	6936.185			−	Streptococcus vestibularis (2.042)	+	−	−
	Intensity (AU)	6861	6271							
	S/N	5	4							
62	m/z	6733.165				−	Enterococcus faecalis (1.884)	+	−	−
	Intensity (AU)	11510								
	S/N	17								
64	m/z			7964.351		−	No reliable identification (1.526)	+	−	−
	Intensity (AU)			9238						
	S/N			3						
74	m/z		6937.344			−	No reliable identification (1.540)	+	−	−
	Intensity (AU)		10112							
	S/N		5							
91	m/z			7964.888		−	Streptococcus gallolyticus (2.197)	+	+	−
	Intensity (AU)			6933						
	S/N			10						
100	m/z	6736.577				−	No reliable identification (1.475)	+	−	+
	Intensity (AU)	7065								
	S/N	4								
126	m/z			7967.395		−	Streptococcus gallolyticus (1.995)	+	−	−
	Intensity (AU)			7655						
	S/N			10						

a “−” and “+” means negative and positive respectively.

Of the 21 samples that were reevaluated as GBS positive on MDAPT, 10 samples were negative on direct MALDI detection but positive on the conventional subculture method. The other 11 samples that tested GBS positive only on MDAPT were considered false positive on MDAPT compared with the culture-based reference method. The discrepancy between the results of the subculture method and those of MDAPT was further evaluated using real-time PCR targeting the *cfb* and *dltS* genes, which were specific to GBS. Among the 11 samples that were GBS positive on MDAPT but negative on subculture, the *cfb* gene was detected in samples 25 and 91, whereas the *dltS* gene in samples 20, 25, and 100 (Table 3).

## DISCUSSION

GBS is one of the leading causes of severe infections, such as sepsis and meningitis, in neonates worldwide. Prevention efforts, such as CDC guidelines and indications, have dramatically decreased the morbidity and mortality of EOD. However, the subculture method, which is considered the gold standard for GBS screening in pregnant women, can produce occasional false-negative results (17, 18). Unfortunately, approximately half of mothers whose neonates developed EOD have tested negative on the subculture method (19–21). Considering this situation, it may be required that false negatives in GBS screening in pregnant women should be reduced as much as possible. In order to improve the sensitivity for GBS screening in pregnant women, optional direct broth testing, such as NAAT or latex agglutination (LA) test, has been developed (11, 12, 22). GBS screening by MALDI-TOF MS using the enrichment broth is also one of unprecedented methods that have the potential to reduce false negatives in GBS screening. Moreover, GBS screening using MALDI-TOF MS can contribute toward a lower workload and shorter turnaround times for microbiology laboratories equipped with it. Therefore, we developed and reported for the first time GBS screening method with MALDI-TOF MS using the broth sample into which vaginal–rectal swab was inoculated.

The rapid BACpro II is a commercial pretreatment kit that uses cationic particles to collect bacteria from positive blood culture bottles more effectively. In previous studies, the rate of bacterial identification directly from blood culture bottles using MALDI-TOF MS was 87.4% with rapid BACpro II assistance, which was significantly higher than the in-house preparation method (57.3%) or the Sepsityper kit (Bruker Daltonics) (68.3%), which is another commercial pretreatment kit (16). In this study, the detection rate of direct MALDI detection with the rapid BACpro II was lower than the conventional subculture method (19.0% versus 27.0%). Furthermore, several bacteria other than GBS were detected in the 10 samples that showed false negative for GBS using the direct MALDI detection alone (Table 2), suggesting that unarranged TOF peaks from various bacteria interfered with the identification of GBS using MALDI-TOF MS. Interestingly, a bacterium that was not detected in the subculture method was identified in direct MALDI detection. For instance, MALDI detection identified *S. anginosus* in one sample, whereas only GBS was detected with the subculture method from the same sample. This indicates that bacterial identification using MALDI-TOF MS may also be influenced by human-derived components that are present in the vaginal–rectal swabs. For example, a previous study found that red blood cells influenced the accuracy of bacterial identification using MALDI-TOF MS (23, 24). MALDI-TOF MS is essentially designed for identifying a bacterium from one colony, not various bacteria. In other words, bacterial identification by MALDI-TOF MS might be influenced by the sample components and presence of multiple bacteria. The reason why the direct detection of pathogenic bacteria using MALDI-TOF MS from a blood culture bottle is successful to some extent may be that, in general, only one bacterial species grows in blood culture. Although the enrichment broth is designed well for the growth of GBS, other Gram-positive cultures may also grow in it. Therefore, direct MALDI detection with the rapid BACpro II was unable to correctly identify GBS with high MALDI score from the enrichment broth.

Several studies have reported the benefit of TOF peaks analysis on bacterial identification using MALDI-TOF MS. For instance, some methods of detecting antimicrobial resistance of pathogenic bacteria based on their TOF peaks have been reported for clinical use, such as those for methicillin-resistant Staphylococcus aureus (25–27), vancomycin-resistant enterococci (28), and carbapenemase-producing *Enterobacteriaceae* (29–32). As for GBS, some S. agalactiae sequence types with high pathogenicity can be distinguished from others by analyzing the presence of the specific protein using MALDI-TOF MS (33, 34). In this study, 10 common TOF peaks specific to GBS were extracted, and four of those, Peak 1 to 4, were adopted to determine the presence of GBS in the broth samples tested. Peak 1 to 4 appeared in more than 70% of the GBS positive samples on the subculture method, whereas they were observed in less than 5% of the GBS negative samples. The other six TOF peaks extracted were recognized in both GBS positive and negative samples. In our previous study (9), bacteria other than GBS which have potential to change the Kyokuto enrichment broth to yellow were the following: E. faecalis (25%), Staphylococcus epidermidis (18%), Corynebacterium amycolatum (6.9%), *S. salivarius* (6.0%), S. oralis (5.2%), and a few other streptococci. Analyzing the TOF peaks of those bacterial strains registered on the Microflex LT database that had at least one or more of Peak 1 to 4 with ≥10% intensities were the following: E. faecalis (0/11), S. epidermidis (0/12), *C. amycolatum* (0/10), *S. salivarius* (3/14), S. oralis (1/38), *S. anginosus* (0/9), S. gallolyticus (0/5), *S. lutetiensis* (0/6), S. mitis (5/39), and *S. parasanguinis* (1/10). Some of the strains erratically showed one GBS-specific TOF peak; however, this did not occur with each bacterial species, and no strains had two or more of Peak 1 to 4. Therefore, we defined the criteria for the “presence of GBS” for MDAPT as follows: at least one or more of Peak 1 to 4, a peak range of within 4 *m/z* or less, and a peak intensity of ≥6,000 AU. Consequently, 34 samples that were GBS positive on the subculture and additional 11 samples that were negative on the subculture met the criteria for the “presence of GBS” (Table 3).

The 11 samples that were GBS positive on MDAPT but negative on subculture were assessed by real-time PCR. Subsequently, the *cfb* gene was detected in samples 25 and 91, whereas the *dltS* gene in samples 20, 25, and 100. In sample 91, the *cfb* gene was detected, but the *dltS* gene was not. This is because the PCR detection limit of the *dltS* gene is higher than that of the *cfb* gene, which is supported by the ≥39 Ct value of sample 91 in the PCR results of the *cfb* gene. On the contrary, only the *dltS* gene was detected in samples 20 and 100. Previous studies reported a CAMP-negative GBS strain, which lacked the *cfb* gene (35, 36); therefore, it can be assumed that samples 20 and 100 might be containing such a strain. NAAT detection of *cfb* and/or *dltS* gene targets indicates that S. agalactiae was likely present in these samples.

More detailed consideration of the criteria for MDAPT may be required, especially depending on the MALDI-TOF MS model available. One of the considerable factors is the signal-to-noise (S/N) ratio, which indicates how much noise the corresponding TOF peak contains. The TOF peak with high S/N ratio has less noise and is valuable for bacterial identification using MALDI-TOF MS (37). If the S/N ratio is added to the identification criteria, some of the samples that showed GBS positive only on MDAPT would be evaluated as “GBS does not exist.” In our study, for instance, if ≥8 S/N ratios were added to the MDAPT criteria, the sensitivity, specificity, PPV, and NPV would be 94.1% (32/34), 93.5% (86/92), 84.2% (32/38), and 97.7% (86/88), respectively. Another factor to consider is the number of corresponding TOF peaks required for identification. If the criteria for MDAPT required at least two or more of Peak 1 to 4, the sensitivity, specificity, PPV, and NPV would be 91.2% (31/34), 98.9% (91/92), 96.9% (31/32), and 96.8% (91/94), respectively. Thus, the addition of other factors, such as the S/N ratio or number of peaks, to the MDAPT criteria may reduce false positives; however, it can also increase false negatives, which may lead to undesirable clinical outcomes. From a clinical point of view, high sensitivity is certainly beneficial for GBS screening in pregnant women for the prevention of severe GBS infectious diseases in neonates. Therefore, we think some false positives can be allowed, but false negatives must not be overlooked. As there is still some room for improvement of the MDAPT criteria, further investigations on the addition of the S/N ratio or selection of TOF peaks specific to GBS should be performed to determine the best conditions for highly accurate GBS screening results with MDAPT.

In our study, we proposed a novel strategy using MALDI-TOF MS for the rapid detection of GBS in pregnant women. GBS detection rates using MALDI detection alone were lower than those with the conventional subculture method; however, the additional analysis of GBS-specific TOF peaks in MDAPT improved the accuracy of GBS detection. In this study, MDAPT demonstrated 100% sensitivity compared with the subculture method. While the specificity of MDAPT compared with subculture was relatively low (88.0%), a portion of false-positive detections may represent the true presence of S. agalactiae, as suggested by its detection on nucleic acid amplification testing. It takes approximately 15 min to identify GBS by direct MALDI detection with the rapid BACpro II and an additional 5 min for TOF peak analysis. In total, MDAPT takes 20 min to perform, which results in shorter turnaround times than those of the subculture method. Optional direct broth testing such as NAAT or LA test has turnaround times within 24 h; however, the accuracy of GBS detection using the LA test varies, and NAAT is costly. Meanwhile, MALDI-TOF MS is widely used, and facilities equipped with it have reasonable running costs. In conclusion, our study indicates that MDAPT can be an alternative method for GBS screening, thereby potentially reducing severe GBS infections in neonates.

## MATERIALS AND METHODS

### Sample collection and ethics.

Based on the CDC guidelines (1), 178 vaginal–rectal specimens were obtained from pregnant women at 35 to 37 gestational weeks from April 1 to October 15, 2019, at Fukushima Medical University Hospital. Each specimen was collected using Transwab (Iwaki, Tokyo, Japan), a transport swab system. Personal information of the specimen providers, such as name, address, age, patient ID, and medical history, were not collected.

The study protocol was approved by the ethics committee of Fukushima Medical University, Fukushima, Japan (Approval No. 2019-260).

### Enrichment broth for GBS.

In this study, the Kyokuto enrichment broth (Kyokuto Pharmaceutical Industrial, Tokyo, Japan) was used to culture GBS from the collected specimen swabs. It is based on Lim broth supplemented with 10 mg/L colistin, 15 mg/L nalidixic acid with the pH indicator bromocresol purple (BCP), and sugar (undisclosed by the manufacturer) 100% metabolizable by S. agalactiae. Therefore, this enrichment broth not only inhibits the growth of Gram-negative bacteria but also indicates that GBS may exist in the broth when the color of the broth changes from purple to yellow after culturing for 24 h or more. If the broth color remains purple or changes to an intermediate color, it is indicated that GBS does not exist in the sample broth, as we have described previously (9).

### Subculture method.

**FIG 1:**
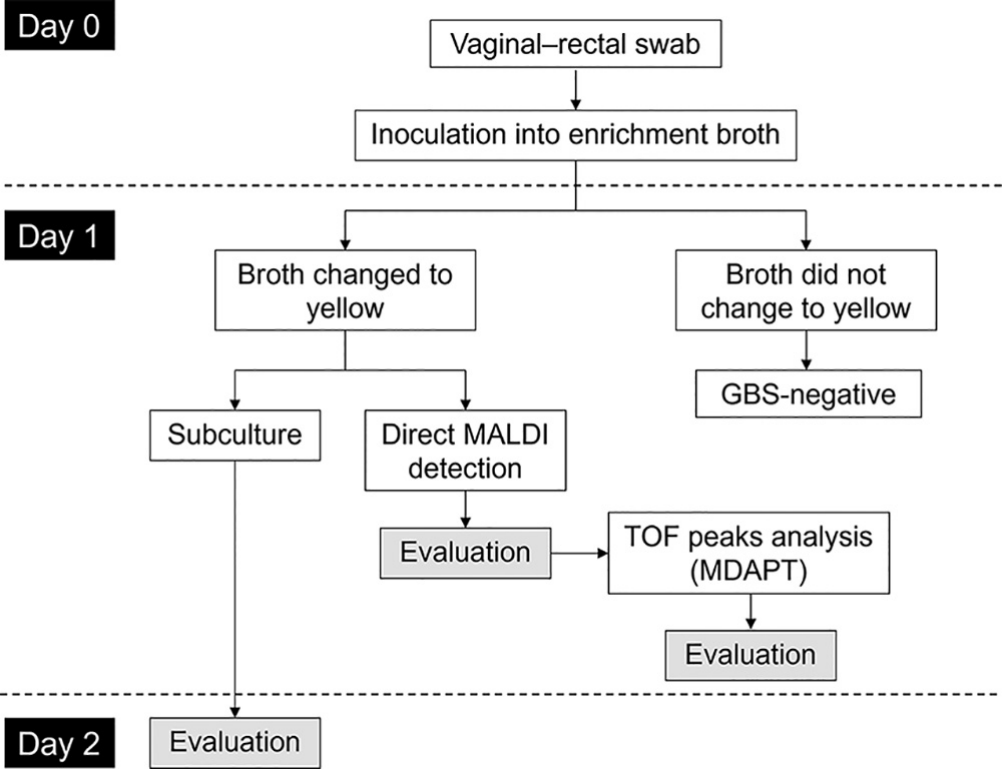
Flowchart of the study methodology. Day 0: the vaginal–rectal swabs were inoculated into the Kyokuto enrichment broth and incubated aerobically for 24 h at 37°C. Day 1: the broths that turned yellow after incubation were both subcultured and tested with direct MALDI detection. The results of direct MALDI detection were then evaluated, and the TOF peaks were analyzed and evaluated (MDAPT). Day 2: subcultured samples were evaluated with MALDI-TOF MS from the isolated colonies.

As shown in Fig. 1, the collected vaginal–rectal specimens were directly inoculated into the Kyokuto enrichment broth and incubated aerobically for 24 h at 37°C. The yellowed broth samples were subcultured aerobically for 24 h at 37°C in 5% sheep blood agar containing colistin and aztreonam (CA agar plate; Becton, Dickinson Japan, Tokyo, Japan), which prevents the growth of Gram-negative bacteria (38). If beta-hemolytic and nonhemolytic GBS-like colonies grew, those were identified to the species level using Microflex LT (Bruker Daltonics, Bremen, Germany). On the contrary, the non-yellowed broth samples were regarded as GBS negative as the manufacturer indicated, which was confirmed by the subculture.

### Direct MALDI detection.

For direct MALDI detection, each yellow-broth sample was pretreated with rapid BACpro II. Then, 200 μL of cationic particles and 200 μL of reaction buffer were added to 800 μL of cultured broth. After centrifugation at 5,000 rpm for 1 min, each precipitate was treated with ethanol/formic acid for extraction. A further 800 μL of ethanol (Fujifilm Wako, Tokyo, Japan) was added, and each precipitate was centrifuged at 5,000 rpm for 1 min. Subsequently, 30 μL of formic acid (Fujifilm Wako) and 30 μL of acetonitrile (Fujifilm Wako) were added to each sample. After centrifugation at 5,000 rpm for 1 min, 1 μL of the supernatant was spotted onto the MALDI-TOF MS target plate and allowed to air dry. Then, 1 μL of alpha-cyano-4-hydroxycinnamic acid matrix solution (HCCA; Bruker Daltonics) was added to each sample plate. After air-drying, the sample was analyzed using the Microflex LT. When GBS was identified with a reliable MALDI score for bacterial species identification (≥2.000), the sample was regarded as GBS positive.

### TOF peaks analysis.

We referred to the database on the Microflex LT software ver. 3.1 (Bruker Daltonics) to determine the TOF peaks common to GBS. On the database, nine GBS were registered: 03 102 CTL, 03 145 CTL, 03 198 CTL, 04 158 CTL, CNR 10 CTL, 16828 DSM, 2134T DSM, 6784 DSM, and V29 CTL. In the nine GBS, several TOF peaks with intensities ≥10% were analyzed (Table S1). We then adopted four TOF peaks that were particularly specific to the nine GBS to determine the presence of GBS: Peak 1 (6,734.41 *m/z*; average intensities of nine GBS [6,729.59–6,736.77]), Peak 2 (6,937.95 *m/z* [6,932.09–6,940.77]), Peak 3 (7,963.55 *m/z* [7,957.35–7,966.28]), and Peak 4 (8,200.27 *m/z* [8,193.93–8,203.04]). Following direct MALDI detection using the Microflex LT, the TOF peaks of each tested sample was analyzed using MATLAB R2019b (MathWorks, Natick, MA, USA), a software for analyzing TOF peaks. A sample was investigated if Peak 1 to 4 were among its TOF peaks. The criteria for GBS positive on the TOF peak analysis were defined as follows: having at least one of Peak 1 to 4, an intensity of 6,000 AU or more, and a tolerance of within 4 *m/z*.

### Nucleic acid amplification test.

From each 500 μL of cultured enrichment broth, DNA was extracted using a QIAamp DNA minikit (Qiagen, Hilden, Germany) according to the manufacturer’s instructions. We adopted real-time PCR for NAAT and targeted the *cfb* and *dltS* genes, which encode the CAMP factor (39, 40) and histidine kinase (41), respectively, and are specific to GBS. The forward and reverse sequences of the primers and appropriate probe for the *cfb* gene were 5′-TTT CAC CAG CTG TAT TAG AAG TA-3′, 5′-GTT CCC TGA ACA TTA TCT TTG AT-3′, and 5′(FAM)-CCC AGC AAA TGG CTC AAA AGC-(BHQ1)3′, respectively. On the contrary, those for the *dltS* gene were 5′-CTG TAA GTC TTT ATC TTT CTC G-3′, 5′-TCC ATT CGC TTA GTC TCC-3′, and 5′ (Eclipse)-ATT TCT ***G***TC TCT A-(FAM)3′, respectively. The TaqMan PCR for the *cfb* gene was 20 μL and prepared as follows: 10 μL of 2x Probe qPCR Mix with UNG (TaKaRa Bio, Tokyo, Japan), 0.4 μL of 10 μM forward and reverse primer, 0.8 μL of 5 μM *cfb* probe, 0.4 μL of 10 μM 50x ROX Reference Dye (TaKaRa Bio) for internal control, 6 μL sterile double-distilled water, and 2 μL of DNA extracted from each sample. The amplification was performed with one cycle at 25°C for 10 min for the UNG activity, followed by one cycle at 95°C for 20 s for initial denaturation, then 40 cycles at 95°C for 1 s and 60°C for 20 s for amplification. The Cycleave PCR for the *dltS* gene (41) was 25 μL, which was prepared as follows: 12.5 μL of 2x CycleavePCR Reaction Mix (TaKaRa Bio), 0.5 μL of 10 μM forward and reverse primer, 1.0 μL of 5 μM *dltS* probe, 0.5 μL of 10 μM 50x ROX Reference Dye for internal control, 8 μL sterile double-distilled water, and 2 μL of DNA extracted from each sample. The amplification was performed with one cycle at 95°C for 2 min for initial denaturation, then 40 cycles at 95°C for 10 s, 50°C for 30 s, and 72°C for 20 s for amplification. Amplification and fluorescent detection were measured by StepOnePlus real-time PCR system (Thermo Fisher Scientific, Waltham, MA, USA). The standard curve was calculated by the 10-fold serial dilutions of DNA from S. agalactiae ATCC 12386. The detection limit of the PCR assay for the *cfb* and *dltS* genes were 500 and 1,000 CFU/assay, respectively. If the threshold cycle (Ct) value was more than 33, amplification of the targeted gene was confirmed by agarose gel electrophoresis.

### Statistical analysis.

Sensitivity, specificity, positive-predictive value (PPV), and negative-predictive value (NPV) were calculated for each GBS detection method by referring to the results of the subculture method. All statistical analyses were performed with IBM SPSS Statistics ver. 25.
