# Supplementary material for: Matrix-Assisted Laser Desorption Ionization–Time-of-Flight Mass Spectrometry with Time-of-Flight Peak Analysis for Rapid and Accurate Detection of Group B Streptococcus in Pregnant Women
Source: Microbiol Spectr. 2022 Apr 18;10(3):e01732-21. doi: 10.1128/spectrum.01732-21 (PMC9241660; doi:10.1128/spectrum.01732-21)
Supplement: SUPPLEMENTAL FILE 1 — Table S1. Download spectrum.01732-21-s001.pdf, PDF file, 0.4 MB [file spectrum.01732-21-s001.pdf]

Supplemental Table 1. Time-of-flight peaks specific to Group B *Streptococcus* extracted from the Microflex LT database

03 102 CTL		03 145 CTL		03 198 CTL		04 158 CTL		CNR 10 CTL		16828 DSM		2134T DSM		6784 DSM		V29 CTL	
m/z [Da]	Intensity [%]	m/z [Da]	Intensity [%]	m/z [Da]	Intensity [%]	m/z [Da]	Intensity [%]	m/z [Da]	Intensity [%]	m/z [Da]	Intensity [%]	m/z [Da]	Intensity [%]	m/z [Da]	Intensity [%]	m/z [Da]	Intensity [%]
**6938.71**[††]	97.89	**6940.10**[††]	100	**6940.77**[††]	100	**6940.34**[††]	100	**6940.24**[††]	100	7527.03	94.13	6884.33	99.89	4451.68	100	**6939.16**[††]	100
4451.03	90.01	3470.60	67.71	3471.03	68.34	3470.71	67.64	3470.77	73.2	4728.26	84.45	4449.44	75.86	5953.03	57.46	4451.4	72.08
5954.17	73.15	4451.52	43.97	4452.03	38.17	4452.03	44.69	4451.95	41.9	7539.84	68.1	5950.44	75.67	**6933.24**[††]	48.73	5379.17	65.24
6248.84	69.83	5955.12	34.87	5955.71	27.2	5955.39	34.64	5955.57	36.35	6127.71	55.92	**7957.35**[##]	42.85	6810.53	45.74	3469.67	62.16
5378.62	69.44	5379.47	34.58	5380.17	26.45	5379.75	33.25	5379.92	27.84	5018.98	54.14	**6932.09**[††]	39.23	6181.36	41.16	5954.70	55.62
3469.4	64.67	**6735.55**[‡‡]	29.97	6842.77	24.25	6842.48	27.17	6184.3	27.84	5952.80	47.65	**6729.59**[‡‡]	38.88	**6733.14**[‡‡]	36.9	6841.27	50.57
6840.81	60.75	6842.19	26.82	6969.96	22.72	**6736.37**[‡‡]	26.03	4103.25	26.9	9455.27	47.19	6178.69	37.09	**7961.51**[##]	31.71	6889.56	46.79
**6734.32**[‡‡]	58.70	6183.79	24.18	**6736.06**[‡‡]	22.61	3421.78	25.42	**6736.77**[‡‡]	25.06	4449.28	46.04	6807.27	35.56	**8198.24**[¶¶]	31.6	**6734.46**[‡‡]	43.97
3420.94	53.81	4102.14	23.04	6893.78	18.94	6184.13	22.07	6842.57	22.6	4100.45	36.21	**8193.93**[¶¶]	32.11	3466.19	26.84	3420.96	41.22
6182.51	45.43	3421.48	23.02	6184.49	18.67	4102.63	20.81	3421.98	22.35	3982.76	34.12	9450.53	22.72	6127.89	26.26	6182.89	39.63
4101.89	44.36	**8202.87**[¶¶]	22.53	6814.58	18.14	**8202.71**[¶¶]	20.34	**8202.21**[¶¶]	21.98	3062.86	31.74	6838.34	21.43	4101.54	23.23	4730.77	36.41
**8200.09**[¶¶]	42.24	3368.37	20.39	3422.28	16.55	3368.60	19.35	3368.81	21.28	3467.1	31.58	5937.2	21.34	6839.97	23.11	6813.01	34.14
6812.88	41.54	6814.07	19.30	**8203.04**[¶¶]	16.47	6814.33	18.64	6892.42	20.94	6181.81	30.62	3443.12	14.15	3982.09	21.08	**7963.96**[##]	33.06
3367.87	39.38	6969.53	18.61	4102.37	15.53	6893.03	17.76	4731.23	17.98	**6936.91**[††]	30.1	3980.33	13.24	3405.91	20.87	**8200.66**[¶¶]	32.74
**7963.56**[##]	31.12	**7965.39**[##]	15.83	3368.78	13.83	6969.56	17.68	6814.48	17.23	**8198.65**[¶¶]	26.43	5375.47	13.23	6888.68	20.67	4101.93	32.26
3407.15	28.52	6893.09	15.6	**7966.28**[##]	11.76	3407.43	14.15	3985.72	16.62	4477.92	22.62	4098.66	11.69	9454.92	20.16	9457.48	31.51
4730.31	27.72	5471.83	14.74	4731.35	11.47	**7965.84**[##]	13.61	6969.14	16.19	**7962.22**[##]	22.58	4485.47	10.74	4728.93	18.47	3983.75	27.86
3983.95	27.37	3985.13	13.93	5472.96	10.27	4731.1	13.5	**7965.82**[##]	15.04	3365.88	21.4			4544.13	17.73	3367.76	27.49
5470.45	27.09	5395.33	12.59			5471.85	12.19	5472.32	14.77	**6733.43**[‡‡]	20.19			5200.08	17.07	3820.66	24.78
3125.37	26.92	4512.00	12.58			3985.19	11.96	3447.26	14.08	3151.1	18.0			5469.04	16.56	3445.23	23.97
4510.87	24.99	3407.48	12.33			4469.1	11.91	4515.51	13.87	3090.31	17.48			5245.86	16.39	5202.27	23.13
9457.39	22.53	4467.79	11.62			3822.82	11.67	3093.5	13.55	7501.2	17.24			3366.71	16.01	7638.66	23.09
5393.51	22.01	5203.32	10.2			4512.48	11.31	3408.36	13.16	5470.17	16.69			5075.32	15.75	3407.37	22.48
4466.34	21.63					9459.55	11.26	9459	12.54	5090.08	15.13			4510.61	15.51	6965.94	21.12
5202.17	20.43					5396.4	11.2	5971.3	11.08	5967.85	14.44			5377.69	14.97	5470.76	20.34
4547.17	19.90					3447.1	10.5	5395.78	10.89	6811.06	14.19			9086.31	14.21	4546.6	19.5
5970.42	18.35					7638.75	10.32	3823.69	10.89	4545.86	13.41			3419.84	13.96	4513.3	18.91
3092.30	17.48							4468.79	10.45	3763.23	13.04			7488.07	11.89	5395.13	17.7
3821.32	16.79							4548.8	10.39	3405.21	12.88			3089.83	10.54	5079.95	16.86
6128.90	16.09							6130.49	10.38	5199.28	12.3			10397.83	10.27	3091.79	15.23
5079.54	15.23									8042.53	12.3			9019.71	10.2	5247.93	14.64
6967.04	14.95									7601.83	11.63			10935.75	10.09	9089.68	13.65
and more										6844.68	10.53					and more	

‡‡; Peak 1, ††; Peak 2, ##; Peak 3, ¶¶; Peak 4
